# Information exchange networks for chronic diseases in primary care practices in Germany: a cross-sectional study

**DOI:** 10.1186/s12875-022-01649-3

**Published:** 2022-03-28

**Authors:** Christine Arnold, Patrick Hennrich, Michel Wensing

**Affiliations:** Department of General Practice and Health Services Research, Heidelberg University Hospital, Im Neuenheimer Feld 130.3, 69120 Heidelberg, Germany

**Keywords:** Information exchange networks, General practice, Social network analysis, Chronic diseases

## Abstract

**Background:**

Coordination of care requires information exchange between health workers. The structure of their information exchange networks may influence the quality and efficiency of healthcare delivery. The aim of this study was to explore and classify information exchange networks in primary care for patients with chronic diseases in Germany.

**Methods:**

A cross-sectional study was carried out between 2019 and 2021. As part of a larger project on coordination of care, this study focused on information exchange in practice teams regarding patients with type 2 diabetes (DM), coronary heart disease (CHD) and chronic heart failure (CHF). Social network analysis was applied to determine the number of connections, density and centralization for each of the health conditions for each of the practices. On the basis of the descriptive findings, we developed typologies of information exchange networks in primary care practices.

**Results:**

We included 153 health workers from 40 practices, of which 25 practices were included in the social network analysis. Four types of information exchange structures were identified for the three chronic diseases: highly connected networks with low hierarchy, medium connected networks with medium hierarchy, medium connected networks with low hierarchy and lowly connected networks. Highly connected networks with low hierarchy were identified most frequently (18 networks for DM, 17 for CHD and 14 for CHF). Of the three chronic conditions, information sharing about patients with DM involved the most team members. Information exchange outside the family practice took place mainly with nurses and pharmacists.

**Conclusions:**

This study identified four types of information exchange structures, which provides a practical tool for management and improvement in primary care. Some practices had few information transfer connections and could hardly be considered a network.

**Trial registration:**

We registered the study prospectively on 7 November 2019 at the German Clinical Trials Register (DRKS, www.drks.de) under ID no. DRKS00019219.

**Supplementary Information:**

The online version contains supplementary material available at 10.1186/s12875-022-01649-3.

## Background

Due to an ageing population and improved acute care, the number of patients with chronic diseases like type 2 diabetes mellitus (DM), coronary heart disease (CHD) and chronic heart failure (CHF) is increasing [1–5]. Many patients receive care from different health workers, who need to coordinate their activities with each other [6, 7]. Well-coordinated healthcare can improve patient outcomes, in terms of a lower risk of hospitalization, fewer days in hospitals, and better controlled blood pressure levels [8, 9]. Primary care has an important role in the delivery and coordination of medical care for these patients [9, 10]. The coordination of healthcare, whether based on team meetings or referral letters, depends on adequate and efficient information exchange between all health workers involved. Inadequate information transfer and poor cooperation may lead to suboptimal treatment of patients [11, 12], reduced continuity of care and patient dissatisfaction with care [6, 13].

In ambulatory care in Germany, the main responsibility for patient care lies with the physicians, with practice assistants involved in delegated procedures (e.g. blood pressure measurement) and administrative tasks [14, 15]. Practice assistants are medical employees who complete 3 years of professional training with emphasis on practice organization and assistance in medical treatment [16]. The coordination of care in primary care practices has been explored in several studies on interprofessional teams, which largely focused on participants’ experiences with aspects of interprofessional collaboration [9, 17]. A complementary perspective is offered by social network analysis (SNA), which is used to identify and examine the factual structure of connections (e.g. based on information transfer) [11, 18–20]. For instance, identification of isolated persons with no connection to other people in the network can reveal gaps in information exchange [21]. Dense networks in which the actors have many connections to one another lead to information reaching the people in the network [22]. Such networks have fewer variations of information pathways, which leads to faster information exchange [23]. High-density networks are associated with fewer hospital days and lower costs [24]. Additionally, the centralization of a network can be examined, which is a measure of the hierarchy in the network. In central networks actors are concentrated around one or more individuals. Central persons take special influence on the exchange of information [25].

The number of studies on networks in primary care is limited. Also, many network studies provide network coefficients (e.g. density) and complex data-analysis, which can be difficult to interpret. Typologies may enhance the interpretation of network data by providing short summaries of typical network structures. In our study, we aimed to document information exchange networks for patients with chronic disease in primary care and to provide a typology of network types.

## Methods

### Study design and study population

This study was part of the ExKoCare project, which examined information exchange networks in German primary care [26]. This three-year project aimed to recruit a sample of 40 GP practices to explore coordination of cardiovascular care in the German states of Baden-Wuerttemberg (approximately 11 million inhabitants, sampling in 10 of the 44 counties), Rhineland-Palatinate (approximately 4 million inhabitants, sampling in 13 of the 36 counties) and Saarland (approximately 1 million inhabitants, sampling in 2 of the 6 counties). The study received ethics approval from the Ethics Committee of the Medical Faculty of Heidelberg (ID: S-726/2018) and from the respective State Medical Chambers. The study population comprised of health workers (physicians, practice assistants and others) who were clustered within 40 primary care practices.

### Data collection

Data on information-exchange inside and outside the practices were collected using a written pseudonymized questionnaire. After the practice owners consented to the ExKoCare study, all health workers were personally contacted and invited *(n = 208)* to participate in the survey. All participants gave written informed consent for the study. Some basic characteristics of the practices were documented in a “practice questionnaire” completed by the practice owner. These characteristics included the size of the practice, as measured by the number of cases per quarter, and the number of team meetings per month.

### Outcomes and measures

#### Constructing social networks by questionnaire

Information exchange networks were measured using personalized questionnaires in a roster format for each practice and participant, following previous studies [27, 28]. The network construction within the practice was assessed using the following instruction: “Please indicate all health workers within your practice with whom you normally exchange information about patients at least once a week. This encompasses counseling and treating individual patients with DM, CHD, and CHF.” The names of the member of the practice were listed and participants had the opportunity to mark each clinical pattern for each person, indicating their connection to another person in the network. Thus, the individual practice members (physicians and practice assistants) formed the nodes or actors of the network, and the connections corresponded to the reported exchange of information about the respective disease. We constructed three networks per practice: one for DM, one for CHD and one for CHF.

#### Network characteristics

As the main outcome of this study, we determined the density of the health worker network within each practice as an indicator of the interconnectedness of health workers with respect to information exchange. The density of the network was defined as the quotient of the number of existing connections and the number of possible connections. In a dense network, information can flow between most or all of the people in the network. Following, so-called network properties are described, which were used in the analysis of the network structures of the practices.

In addition, a number of secondary outcomes were documented: *Number of connections* refers to the number of absolute existing connections in the network. *Centralization* indicates how strongly a network is centered around a single person. In a network with high centralization, information flows predominantly via the central person. *Reciprocity* indicates the number of returned relationships in a directed network. We used it to confirm the validity of the information exchange in the network. All four network properties were calculated separately for each disease.

##### Descriptive variables

Descriptive measures included practice size (number of staff members); type of practice (single-handed practice, group practice, shared practice and ambulatory health care centre); cases per quarter (< 500 cases, 500–1000 cases, 1001–1500 cases and > 1500 cases), documentation (digitally, paper-based, digitally and paper-based); presence of a case manager (yes or no), number of team meeting per quarter, number of cardiologists with whom information is exchanged once at least once a month.

Information exchange outside the practice was measured with the following instruction: “Please indicate the occupational groups outside of your practice with whom you exchange information at least once a week regarding patients with DM, CHD, and CHF. This exchange encompasses counselling and treating individual patients through various means of communication (e.g., prescriptions, letters, phone calls).” After this instruction, different occupational groups were listed, including pharmacists, nutritionists, physiotherapists, nursing home nurses, nurses from ambulatory nursing services, rehabilitation exercise classes, classes for cardiology-related exercises, rehabilitation centres, physicians’ assistants outside of the practice, psychologists, respiratory physicians, internists (all fields except cardiology) and occupational health physicians.

### Data analysis

Descriptive analyses were conducted to determine the practices characteristics and information exchange outside the general practices, including frequencies and means with standard deviation (SD). To assess differences between subgroups, we performed the Wilcoxon-test, Friedman-test or Chi^2^-test, depending on the distribution of the variables and the scale level of the variables.

Next, we performed a social network analysis with the statistic software R version 4.0.2 using RStudio version 1.2.5033 (igraph package) to represent the exchange of information within each primary care practice. To validate the information exchange connections, we measured the reciprocity of the networks, using directed networks. If the reciprocity was above 0.6, missing values were reconstructed by creating undirected networks [29].

In the third phase a typology of information exchange networks was developed. We plotted the networks for each primary care practice. In the plots, the connections between health workers represent the weekly exchange of information about patients with the respective condition. In an explorative process, we decided (post-hoc) to focus on density and centralization for establishing network types.

In the final phase, we included networks with at least three people and at most one missing value (non-response from a practice member); otherwise, information about the connections would have been lost and the network would not have been representative for this practice. In the case of one missing value, a reconstruction was performed according to scientific recommendations [29, 30].

To compare the undirected networks for the three diseases, we calculated the mean with SD and median with interquartile range of the number of connections, density, and centralization of the networks and compared them using the Friedman-test. Significance level of all analyses was set at α = 0.05. Since no classification was found in the literature to identify network typologies, we used a data-driven approach to derive types of networks. After visual inspection of network plots, we classified the networks into 3 groups (low ≤0.33, medium > 0.33–0.67 and high > 0.67) according to the density and centralization separately and then into combined types. Density and centralization are negatively correlated by definition. For example, in a dense network with density of 1 where all actors are interconnected, the centralization value is 0. Nevertheless, at medium levels of density, values for centralization can vary.

## Results

### Description of study population

Between November 2019 and January 2021, we received a total of 153 questionnaires from 40 practices (73.6% response rate for individuals and 95.2% for practices). The recruitment period was extended for 6 months due to the then ongoing COVID-19 pandemic. Within the 40 practices, 47 physicians and 106 practice assistants participated. For social network analysis, we excluded practices with more than one non-responding practice member *(n = 9)* and fewer than three individuals *(n = 6)*, leaving 25 practices with a total of 121 health workers, for which we had 108 completed questionnaires (response rate 89.3% for individuals).

Table 1 presents the characteristics of all the practices and the 25 practices included in the social network analysis. On average (mean), there were 5.0 (SD 2.53) health workers per practice. Most practices (32 (80.0%) were organized as single-handed practices.

**Table 1:** Practice characteristics

	All practices *n* (%) *N* = 40	Practices included in SNA *n* (%) *N* = 25
**Number of health workers per practice** mean (SD), range	5.00 (2.53), range 2–15	4.84 (1.31), range 3–7
**Number of assistants** mean (SD), range	3.63 (2.05), range 1–12	3.52 (1.05), range 2–6
**Number of physicians** mean (SD), range	1.37 (0.66), range 1–4	1.32 (0.48), range 1–2
**Type of practice**	*n = 40*	*n = 25*
Single-handed practice	32 (80.0)	20 (80.0)
Group practice	7 (17.5)	5 (20.0)
Shared practice	1 (2.5)	0 (0.0)
Ambulatory health care centre	0 (0.0)	0 (0.0)
**Cases per quarter**	*n = 38*	*n = 24*
< 500 cases	3 (7.9)	1 (4.2)
500–1000 cases	13 (34.2)	8 (33.3)
1001–1500 cases	15 (39.5)	11 (45.8)
> 1500 cases	7 (18.4)	4 (16.7)
**Documentation**	*n = 39*	*n = 25*
Digitally	17 (43.6)	11 (44.0)
Paper-based	0 (0.0)	0 (0.0)
Digitally and paper-based	22 (56.4)	14 (56.0)
**Case management**	*n = 39*	*n = 24*
No	25 (64.1)	17 (70.8)
Yes	14 (35.9)	7 (29.2)
**Team meetings per 3 months**	*n = 39*	*n = 24*
No meeting	5 (12.8)	3 (12.5)
1 meeting	17 (43.6)	10 (41.7)
2 meetings	8 (20.5)	6 (25.0)
3 meetings	6 (15.4)	2 (8.3)
4 meetings	0 (0.0)	0 (0.0)
> 4 meetings	3 (7.7)	3 (12.5)

*SNA* Social network analysis

*SD* Standard deviation

### Information exchange with health workers outside the practice

In the analysis of information exchange with health workers outside the practices, all 153 questionnaires from the health workers were included. Additional file 1 provides detailed information on information exchange between different professional groups outside the primary care practices and the members of the practices. About half of the health professionals exchanged information about the three types of patients with pharmacists, nursing home nurses, and ambulatory nursing services once a week. Most of them mainly exchanged information with pharmacists, nursing home nurses, and ambulatory nursing services. Less than 20% of the practice members stated that they exchange information with the other professional groups. Comparing physicians and practice assistants, more practice assistants reported sharing information. More physicians than practice assistants only exchanged information with internists and rehabilitation centres. No regular information was exchanged with occupational physicians.

Aggregated at practice level, information on CHD was exchanged with a mean of 2.91 (SD 1.29, range 0–5) cardiologists. Physicians exchanged information with a mean of 3.93 (SD 1.85) and non-physicians with a mean of 2.21 (SD 1.63) cardiologists per month. For CHF, on practice level, there was a mean of 2.66 (SD 1.21, range 0–5), for physicians a mean of 3.65 (SD 1.96) and for non-physicians mean of 1.99 (SD 1.54) cardiologists.

### Description of information exchange networks within the practice

With regard to the validation of information exchange, overall reciprocity was 0.70, so we postulated undirected networks in the further analysis. Considering the networks of the different diseases, those for DM had the most connections, with a total of 201 connections across the 25 practices. The networks for CHD had 191 connections, and those for CHF had 183. Table 2 presents the network characteristics of the 25 included practices divided according to DM, CHD and CHF. Across all practices, the networks for DM had the highest density. The mean densities were 0.82 (SD 0.21, range 0.20–1.00) for DM, 0.79 (SD 0.24, range 0.13–1.00) for CHD and 0.76 (SD 0.25, range 0.07–1.00) for CHF (Friedman chi^2^ = 8.22, df = 2, *p* = 0.02).

**Table 2:** Network characteristics for type 2 diabetes, coronary heart disease and heart failure

	Type 2 diabetes Mean (SD) Median (IQR)	Coronary heart disease Mean (SD) Median (IQR)	Chronic heart failure Mean (SD) Median (IQR)	*p*-value
Reciprocity	0.78 (0.38)	0.72 (0.42)	0.59 (0.45)	
	1.00 (0.78–1.00)	1.00 (0.67–1.00)	0.79 (0.00–1.00)	0.03^*^
Number of connections	8.04 (4.96)	7.64 (4.76)	7.32 (4.60)	
	8.00 (3–10)	8.00 (3–10)	8.00 (3–10)	0.02^*^
Density	0.82 (0.21)	0.79 (0.24)	0.76 (0.25)	
	0.90 (0.67–1.00)	0.90 (0.67–1.00)	0.80 (0.62–1.00)	0.02^*^
Centralization	0.16 (0.16)	0.16 (0.17)	0.18 (0.18)	
	0.10 (0.00–0.33)	0.10 (0.00–0.33)	0.13 (0.00–1.00)	0.59

^*^ *p*-value ≤0.05 statistically significant tested by Friedman-test

*SD* standard deviation, *IQR* Interquartile range

### Typology

Table 3 offers an overview of the number of network types classified by density and centralization which were found independent of network size in the sample. Of the 25 practices, 18 were highly connected for DM, 17 for CHD and 14 for CHF. Regarding centralization, most networks had low values: 20 for DM, 22 for coronary heart disease, and 21 for chronic heart failure. Overall, density and centralization were strongly negatively correlated with each other (e.g., the correlation between density and centralization in the DM networks was rho = − 0.91, *p* < 0.01).

**Table 3:** Overview of the numbers of identified networks classified by density and centralization

	Type 2 diabetes *n* (%) *n* = 25	Coronary heart disease *n* (%) *n* = 25	Chronic heart failure *n* (%) *n* = 25
**Classification by density**			
Highly connected	18 (72.0)	17 (68.0)	14 (56.0)
Medium connected	6 (24.0)	7 (28.0)	10 (40.0)
Lowly connected	1 (4.0)	1 (4.0)	1 (4.0)
**Classification by centralization**			
Highly centralized	0 (0.0)	0 (0.0)	0 (0.0)
Medium centralized	5 (20.0)	3 (12.0)	4 (16.0)
Lowly centralized	20 (80.0)	22 (88.0)	21 (84.0)

According to our chosen classification of density and centralization, we identified 4 network types in the data: type A: highly connected networks (with logically low hierarchy), of which fully connected networks are a special case; type B: medium connected with medium hierarchy (high hierarchy in combination with a medium connection was not identified in the data, the highest centralization value of a practice was 0.63); type C: medium connected networks with low hierarchy; type D: lowly connected with low hierarchy (as a logical consequence) – rare in these data.

**Fig. 1:**
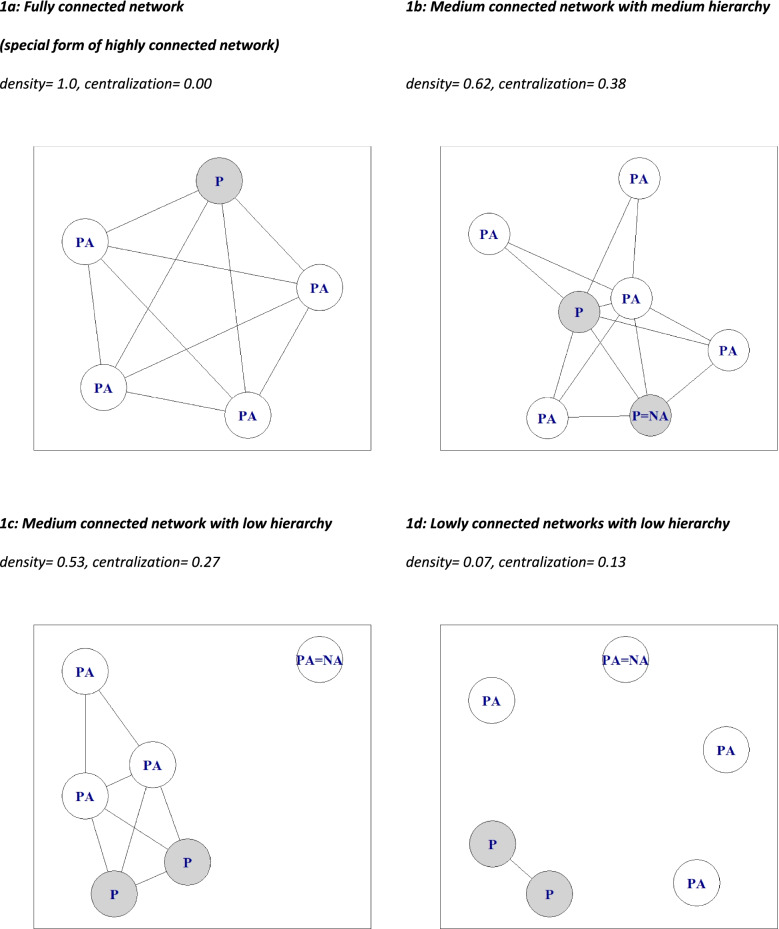
Information exchange networks for CHD and CHF. *P* physician, *PA* practice assistant, *P* NA: non-response physician, *PA* NA: non-response practice assistant. The circles marked “P” indicate physicians, those marked “PA” indicate practice assistants and the lines between them indicate information change once per week

Data-based examples of different types of networks are presented in Fig. 1. Figure 1 a shows a fully connected network. Information was most frequently exchanged between all practice members, indicating a network density of 1. Density of one is equivalent to a centralization of 0, since all members of the network have the same number of connections and thus no actor is centrally located. Fully connected networks existed in 10 cases of CHD networks, 9 of DM and 9 of CHF. The negative correlation of density and centralization was also shown in type B and C: medium connected team, in which the values of density and centrality converged. Low-connected teams were characterized by equally low density and centrality, this was only the case in one network per disease (see Fig. 1 d). In this network, no practice assistants were included in the information exchange.

## Discussion

Four types of information exchange structures were identified for the three chronic diseases: highly connected networks with low hierarchy, medium connected networks with medium hierarchy, medium connected networks with low hierarchy and lowly connected networks. Of the three chronic conditions, information sharing about patients with DM involved the most team members. Information exchange outside the practice took place mainly among nurses and pharmacists.

German primary care practices tend to be small as compared to those in other countries, such as the United Kingdom. In addition to the physicians, there are usually only practice assistants in the family practice [31]. Due to differences across health care systems, generalization of the findings may be questionable. Nevertheless, the findings reflected those of similar studies in other countries. For instance, the finding that the highest level of information exchange concerned DM patients is consistent with the findings of a study in The Netherlands [28], who found that, in primary care, information sharing in the care of patients with DM is higher than in care for patients with CHD or chronic obstructive pulmonary disease. The higher level of information sharing about patients with DM is probably explained by the development of disease-management programmes. In Germany, a programme for type 2 diabetes has been in place since 2002, whereas the programme for CHD was implemented as recently as 2019. The disease-management programmes ensure that the practice assistants are more integrated into patient care [32]. Although even the heart failure guidelines call for interprofessional care for these patients suggests our analysis that such interprofessional care is less implemented for cases of CHF than for cases of DM [33].

Fully connected networks were most prevalent in our study; these have by definition low hierarchy. Information flows faster in dense networks, which can enhance the coordination of care for individual patients and enhance the uptake of recommended clinical practices generally. Both can improve the quality and outcomes of healthcare. In their social network study, Mundt et al. [24] found that high network density was associated with a 38% reduction in hospital days. Similar results were obtained by Kuo et al. [34], who presented positive correlation between network density and hospitalization in the context of COPD, HF, and DM.

Furthermore, Espinoza et al. [35] showed that SNA can be used to identify structures of the teams and to provide the correlation with the team satisfaction. A maximum of network density of one was associated with higher team satisfaction. Subgroups and isolated individuals were found in networks with low density and low team satisfaction.

Interestingly, maximum centralization in the information exchange networks (e.g. a star structure) was not found. This reflects the intensive communication between health workers in most primary care practices, who largely function as patient care teams. Lower centralization in information exchange networks in primary care practices may reflect better teamwork [36], but it may come with a higher number of connections that is not optimally efficient (as information exchange requires effort). Primary care practices with medium connections and low hierarchy may balance teamwork and efficiency of communication between health workers.

Regarding information exchange with health workers outside the practices, we showed that information flows primarily to and from pharmacists and nurses. Information exchange with pharmacists can be explained by the fact that the crucial treatment of the diseases studied is drug therapy. General practitioners are repeatedly faced with the challenge of adequate medication management due to polypharmacy [37]. Interventions integrating pharmacists into care for patients with chronic conditions can improve outcomes for them [38, 39]. The information exchange with cardiologists was measured separately and is not considered in this discussion.

Due to the increase in multimorbidity, the need for care is increasing [2, 40]. Care is carried out by nurses in both inpatient facilities and outpatient services. Since nurses in Germany are dependent on written prescriptions from physicians, a regular communication is essential here, which may explain the weekly information exchange.

Social network analysis has proven to be an analysis tool for identifying structures in German primary care practices. This can be used to improve communication and teamwork [11]. It could be explored that practice assistants are more involved in the treatment of patients with DM than in patients with heart failure. Existing structures in the field of DM should be used to promote the treatment of patients with heart failure. It can be assumed that information regarding DM reaches all team members in the family practice faster compared to information regarding heart failure. In addition, further studies should examine how these team structures relate to team satisfaction and patient outcomes.

### Strengths and limitations

This is one of the first social network studies which developed typologies of networks in German primary care, with a good response rate of health workers in the participating practices. As social network studies are sensitive to non-response, however, we could only include 25 of 40 practices in the main analyses. We decided against imputation of missing values for more than one missing, because we felt that the networks were too small for this. Despite these limitations, the study revealed substantial variation regarding information exchange networks. Our study reports the number of connections in the networks, not the quality of information exchange. Furthermore, we did not examine the impact on the quality and outcomes of care.

## Conclusions

The collaboration of health professionals in primary care practices is central for chronic illness care. This study identified four types of information exchange structures, which provides a practical tool for management and improvement in primary care. We also recognized, however, that some practices could not be considered a team or network due to the low number of connections. Furthermore, in such analyses, non-respondents remain a challenge and appropriate procedures need to be developed to be able to use as much data as possible. Building on the knowledge gained about information-sharing structures, our results can be used to develop strategies for implementing innovations or guidelines to promote quality of care.

## Supplementary Information

**Additional file 1: Table S1.** Information exchange outside the general practitioner’s practice. **Table S2.** Reporting Guideline.

## Data Availability

The datasets analysed during the current study are available from the corresponding author on reasonable request.

## Abbreviations

CHD: Coronary heart disease
CHF: Chronic heart failure
COVID-19: Coronavirus disease 2019
Df: Degree of freedom
DM: Type 2 diabetes mellitus
GP: General practitioner
IQR: Interquartile range
P: Physician
PA: Practice assistant
SD: Standard deviation
SNA: Social network analysis
