# Supplementary material for: Information exchange networks for chronic diseases in primary care practices in Germany: a cross-sectional study
Source: BMC Prim Care. 2022 Mar 28;23:56. doi: 10.1186/s12875-022-01649-3 (PMC8958478; doi:10.1186/s12875-022-01649-3)
Supplement: Supplementary file 1 — Additional file 1: Table S1. Information exchange outside the general practitioner’s practice. Table S2. Reporting Guideline. [file 12875_2022_1649_MOESM1_ESM.docx]

**Additional file 1a:** Information exchange outside the general practitioner’s practice (N=153, physicians n=47, physicians assistants (n=106)

|  | **Type 2 diabetes** | | **Coronary heart disease** | | **Chronic heart failure** | |
| --- | --- | --- | --- | --- | --- | --- |
|  | No  N (%) | Yes  N (%) | No  N (%) | Yes  N (%) | No  N (%) | Yes  N (%) |
| **Pharmacists**  Physicians’ assistants  Physicians | 61 (39.9)  33 (31.1)  28 (59.6) | 92 (60.1)  73 (68.9)  19 (40.4) | 75 (49.0)  44 (41.5)  31 (66.0) | 78 (51.0)  62 (58.5)  16 (34.0) | 79 (51.6)  47 (44.3)  32 (68.1) | 74 (48.4)  59 (55.7)  15 (31.9) |
| **Nutritionists**  Physicians’ assistants  Physicians | 145 (94.8)  99 (93.4)  46 (97.9) | 8 (5.2)  7 (6.6)  1 (2.1) | 151 (98.7)  104 (98.1)  47 (100) | 2 (1.3)  2 (1.9)  0 (0.0) | 151 (98.7)  104 (98.1)  47 (100) | 2 (1.3)  2 (1.9)  0 (0.0) |
| **Physiotherapists**  Physicians’ assistants  Physicians | 141 (92.2)  96 (90.6)  45 (95.7) | 12 (7.8)  10 (9.4)  2 (4.3) | 142 (92.8)  95 (89.6)  47 (100) | 11 (7.2)  11 (10.4)  0 (0.0) | 146 (96.0)  99 (93.4)  47 (100) | 7 (4.0)  7 (6.6)  0 (0.0) |
| **Nurses in a nursing home**  Physicians’ assistants  Physicians | 65 (42.5)  41 (38.7)  24 (51.1) | 88 (57.5)  65 (61.3)  23 (48.9) | 76 (49.7)  48 (45.3)  28 (59.6) | 77 (50.3)  58 (54.7)  19 (40.4) | 73 (47.7)  48 (45.3)  25 (53.2) | 80 (52.3)  58 (54.7)  22 (46.8) |
| **Ambulatory nursing services**  Physicians’ assistants  Physicians | 57 (37.3)  32 (30.2)  25 (53.2) | 96 (62.7)  74 (69.8)  22 (46.8) | 73 (47.7)  42 (39.6)  31 (66.0) | 80 (52.3)  64 (60.4)  16 (34.0) | 75 (49.0)  44 (41.5)  31 (66.0) | 78 (51.0)  62 (58.5)  16 (34.0) |
| **Rehabilitation exercise classes**  Physicians’ assistants  Physicians | 149 (97.4)  103 (97.2)  46 (97.9) | 4 (2.6)  3 (2.8)  1 (2.1) | 148 (96.7)  102 (96.2)  46 (97.9) | 5 (3.3)  4 (3.8)  1 (2.1) | 150 (98.0)  103 (97.2)  47 (100) | 3 (2.0)  3 (2.8)  0 (0.0) |
| **Classes for cardiology-related**  **exercises**  Physicians’ assistants  Physicians | 153 (100)  106 (100)  47 (100) | 0 (0)  0 (0)  0 (0) | 147 (96.1)  102 (96.2)  45 (95.7) | 6 (3.9)  4 (3.8)  2 (4.3) | 149 (97.4)  103 (97.2)  46 (97.9) | 4 (2.6)  3 (2.8)  1 (2.1) |

**Additional file 1b:** Information exchange outside the general practitioner’s practice

|  | **Type 2 diabetes** | | | | **Coronary heart disease** | | | | **Chronic heart failure** | | | |
| --- | --- | --- | --- | --- | --- | --- | --- | --- | --- | --- | --- | --- |
|  | No  N (%) | | Yes  N (%) | | No  N (%) | | Yes  N (%) | | No  N (%) | | Yes  N (%) | |
| **Rehabilitation centres**  Physicians’ assistants  Physicians | 147 (96.1)  102 (96.2)  45 (95.7) | | 6 (3.9)  4 (3.8)  2 (4.3) | | 147 (96.1)  102 (96.2)  45 (95.7) | | 6 (3.9)  4 (3.8)  2 (4.3) | | 147 (96.1)  102 (96.2)  45 (95.7) | | 6 (3.9)  4 (3.8)  2 (4.3) | |
| **Physicians’ outside of your practice**  Physicians’ assistants  Physicians | | 136 (88.9)  93 (87.7)  43 (91.5) | | 17 (11.1)  13 (12.3)  4 (8.5) | | 137 (89.5)  95 (89.6)  42 (89.4) | | 16 (10.5)  11 (10.4)  5 (10.6) | | 138 (90.2)  96 (90.6)  42 (89.4) | | 15 (9.8)  10 (9.4)  5 (10.6) |
| **Physicians’ assistants**  **outside the practice**  Physicians’ assistants  Physicians | 127 (83.0)  84 (79.3)  43 (91.5) | | 26 (17.0)  22 (20.8)  4 (8.5) | | 133 (86.9)  89 (84.0)  44 (93.6) | | 20 (13.1)  17 (16.0)  3 (6.4) | | 133 (86.9)  89 (84.0)  44 (93.6) | | 20 (13.1)  17 (16.0)  3 (6.4) | |
| **Psychologists**  Physicians’ assistants  Physicians | 149 (97.4)  103 (97.2)  46 (97.9) | | 4 (2.6)  3 (2.8)  1 (2.1) | | 150 (98.0)  103 (97.2)  47 (100) | | 3 (2.0)  3 (2.8)  0 (0) | | 151 (98.7)  104 (98.1)  47 (100) | | 2 (1.3)  2 (1.9)  0 (0.0) | |
| **Respiratory physicians**  Physicians’ assistants  Physicians | 149 (97.4)  102 (96.2)  47 (100) | | 4 (2.6)  4 (3.8)  0 (0.0) | | 146 (95.4)  100 (94.3)  46 (97.9) | | 7 (4.6)  6 (5.7)  1 (2.1) | | 145 (94.8)  99 (93.4)  46 (97.9) | | 8 (5.2)  7 (6.6)  1 (2.1) | |
| **Internists**  *(all fields except cardiology)*  Physicians’ assistants  Physicians | 127 (83.0)  89 (84.0)  38 (80.9) | | 26 (17.0)  17 (16.0)  9 (19.2) | | 127 (83.0)  89 (84.0)  38 (80.9) | | 26 (17.0)  17 (16.0)  9 (19.1) | | 131 (85.6)  90 (84.9)  41 (87.2) | | 22 (14.4)  16 (15.1)  6 (12.8) | |
| **Occupational Medicine**  Physicians’ assistants  Physicians | 153 (100)  106 (100)  47 (100) | | 0 (0.0)  0 (0.0)  0 (0.0) | | 153 (100)  106 (100)  47 (100) | | 0 (0.0)  0 (0.0)  0 (0.0) | | 153 (100)  106 (100)  47 (100) | | 0 (0.0)  0 (0.0)  0 (0.0) | |
